# Cognitive enhancing effect of rTMS combined with tDCS in patients with major depressive disorder: a double-blind, randomized, sham-controlled study

**DOI:** 10.1186/s12916-024-03443-7

**Published:** 2024-06-20

**Authors:** Xingxing Li, Junyao Liu, Shuochi Wei, Chang Yu, Dongmei Wang, Yuchen Li, Jiaxin Li, Wenhao Zhuang, Rui-Chen-Xi Luo, Yanli Li, Zhiwang Liu, Yuqiu Su, Jimeng Liu, Yongming Xu, Jialin Fan, Guidong Zhu, Weiqian Xu, Yiping Tang, Hui Yan, Raymond Y. Cho, Thomas R. Kosten, Dongsheng Zhou, Xiangyang Zhang

**Affiliations:** 1 Ningbo Key Laboratory for Physical Diagnosis and Treatment of Mental and Psychological Disorders, Affiliated Kangning Hospital of Ningbo University (Ningbo Kangning Hospital), Ningbo, Zhejiang China; 2 CAS Key Laboratory of Mental Health, Institute of Psychology, Chinese Academy of Sciences, Beijing, China; 3 Department of Psychology, University of Chinese Academy of Sciences, Beijing, China; 4 The Second People’s Hospital of Lishui, Lishui, Zhejiang China; 5 Taizhou Second People’s Hospital, Taizhou, Zhejiang China; 6 Department of Psychiatry and Behavioral Sciences, Baylor College of Medicine, Houston, TX USA

**Keywords:** Major depressive disorder, rTMS, tDCS, Clinical trial, Cognition

## Abstract

**Background:**

Cognitive dysfunction is one of the common symptoms in patients with major depressive disorder (MDD). Repetitive transcranial magnetic stimulation (rTMS) and transcranial direct current stimulation (tDCS) have been studied separately in the treatment of cognitive dysfunction in MDD patients. We aimed to investigate the effectiveness and safety of rTMS combined with tDCS as a new therapy to improve neurocognitive impairment in MDD patients.

**Methods:**

In this brief 2-week, double-blind, randomized, and sham-controlled trial, a total of 550 patients were screened, and 240 MDD inpatients were randomized into four groups (active rTMS + active tDCS, active rTMS + sham tDCS, sham rTMS + active tDCS, sham rTMS + sham tDCS). Finally, 203 patients completed the study and received 10 treatment sessions over a 2-week period. The Repeatable Battery for the Assessment of Neuropsychological Status (RBANS) was performed to assess patients’ cognitive function at baseline and week 2. Also, we applied the 24-item Hamilton Depression Rating Scale (HDRS-24) to assess patients’ depressive symptoms at baseline and week 2.

**Results:**

After 10 sessions of treatment, the rTMS combined with the tDCS group showed more significant improvements in the RBANS total score, immediate memory, and visuospatial/constructional index score (all *p* < 0.05). Moreover, post hoc tests revealed a significant increase in the RBANS total score and Visuospatial/Constructional in the combined treatment group compared to the other three groups but in the immediate memory, the combined treatment group only showed a better improvement than the sham group. The results also showed the RBANS total score increased significantly higher in the active rTMS group compared with the sham group. However, rTMS or tDCS alone was not superior to the sham group in terms of other cognitive performance. In addition, the rTMS combined with the tDCS group showed a greater reduction in HDRS-24 total score and a better depression response rate than the other three groups.

**Conclusions:**

rTMS combined with tDCS treatment is more effective than any single intervention in treating cognitive dysfunction and depressive symptoms in MDD patients.

**Trial registration:**

Chinese Clinical Trial Registry (ChiCTR2100052122).

## Background

Major depressive disorder (MDD) is a recurrent and severe mental disease [1], and cognitive impairments in attention, executive functioning, and immediate memory are the most frequent symptoms of MDD patients, which impair their psychosocial function and limit their quality of life [2]. Most antidepressants do not improve any cognitive impairments in MDD patients [3, 4]. Therefore, there is an urgent need to develop and optimize novel therapies for cognitive impairment in MDD patients.

Repetitive transcranial magnetic stimulation (rTMS) is considered a safe and effective treatment for MDD patients [5], but its cognition-enhancing effect in MDD patients remained inconclusive. Meta studies have shown that rTMS applied to the prefrontal cortex for patients with neuropsychiatric conditions does not result in robust cognitive enhancing effects but other studies showed a moderate improvement in psychomotor speed and cognitive control ability [6, 7]. Recent studies have shown that rTMS can also enhance verbal memory in patients with treatment-resistant depression [8].

According to a meta-study [9], therapeutic transcranial direct current stimulation (tDCS) to the dorsolateral prefrontal cortex (DLPFC) in different diseases, such as depression and schizophrenia, can produce part cognitive benefits like attention/vigilance, working memory, and failure in executive functioning, processing speed, verbal fluency, verbal learning, and social cognition. However, another recent meta-study has reported that these improvements are dependent on improvements in mood [10]. Other studies on the effects of tDCS in patients with schizophrenia [11] and Alzheimer’s disease [12] also support the efficacy of tDCS can improve cognitive performance on certain sub-dimensions.

Because tDCS alters neuronal resting membrane potentials, whereas rTMS generates neuronal action potentials, several studies have found more lasting changes in cortical excitability and plasticity when tDCS is used as a pre-stimulus followed by rTMS [13, 14]. Furthermore, several studies have investigated the effects of rTMS combined with tDCS in healthy individuals or in patients with stroke and Parkinson’s disease, and have shown that rTMS combined with tDCS is superior to rTMS alone in terms of improving motor function and can have a more positive effect on cortical plasticity [15, 16]. Overall, both rTMS and tDCS produce cognitive benefits, and combining these two interventions appears to produce synergistic effects. Furthermore, the current hot topic and challenge in clinical research is how to shorten the onset of efficacy. Classical intervention cycles are usually 4–6 weeks, but some depressed patients have poor compliance due to the long duration of the intervention. In addition, there are other objective reasons, such as the necessity for patients to receive stimulation in a fixed location, resulting in many patients not being able to check in and complete an adequate course of intervention. Conclusions about whether shorter intervention cycles could produce a cognition improvement are inconsistent. Therefore, we investigated the potential benefits of combining rTMS with tDCS on cognitive improvement through a randomized controlled clinical trial by comparing rTMS, tDCS, rTMS with tDCS, and both two sham stimulations. We hypothesized that the combination of tDCS with rTMS would be more effective than other treatments. In addition, we examined depressive symptoms and response rates after treatment and evaluated the side effects and safety of the combination of rTMS and tDCS.

## Methods

### Participants

The study participants were recruited from inpatients at Ningbo Kangning Hospital, Lishui Second People's Hospital, and Taizhou Second People's Hospital. All patients were diagnosed with MDD in the Diagnostic and Statistical Manual of Mental Disorders-V (DSM-V) and were assessed by the Mini-International Neuropsychiatric Interview. Other inclusion criteria included (1) 18 to 65 years of age; (2) ≥ 20 on the 24-item Hamilton Depression Rating Scale (HDRS-24), and (3) taking stable antidepressants.

Exclusion criteria were as follows: (1) a lifetime history of any other psychiatric disorders or severe brain injury; (2) history of electroconvulsive therapy, TMS, tDCS, transcranial alternating current stimulation, or other neurostimulation treatments within the past 3 months; and (3) contraindications to magnetic fields, such as epilepsy, cardiovascular complications, or metallic implants in the head.

Criteria for loss of participants were (1) discontinuation from the efficacy analysis after 2 consecutive or more than 2 cumulative treatment failures; (2) change in medications during the 2-week trial period; and (3) development of serious adverse effects during treatment.

The Ethics Committee of Ningbo Kangning Hospital approved the study, and all participants signed a written informed consent prior to the study. The study was registered with the Chinese Clinical Trial Registry (ChiCTR2100052122) at http://www.chictr.org.cn.

### Randomization and rTMS or tDCS treatment and blinding

**Fig. 1:**
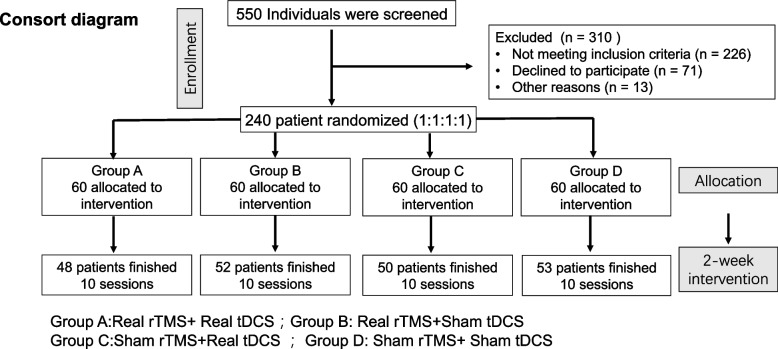
The CONSORT diagram of the primary phases of the clinical trial

The CONSORT chart for this clinical trial is shown in Fig. 1. Patients were randomly assigned to one of the four groups by an impartial third party using a computer-generated randomization list created using basic randomization with equal odds (group A: active rTMS + active tDCS, group B: active rTMS + sham tDCS, group C: sham rTMS + active tDCS, group D: sham rTMS + sham tDCS). After enrollment, patients received stable SSRI antidepressant medication, and only one-time dosage was adjusted during the 2 weeks of hospitalization without changing the type of medication. The medication of the patients in each group is detailed in Table 1, and there was no statistically significant difference between the four groups.

**Table 1:** Demographic and clinical characteristics of the patients

	Real rTMS + real tDCS (*N* = 60)	Real rTMS + sham tDCS (*N* = 60)	Sham rTMS + real tDCS (*N* = 60)	Sham rTMS + sham tDCS (*N* = 60)	*F*/*χ* ^2^	*P* value
Age (years)	31.00 ± 15.12	30.37 ± 12.30	31.43 ± 14.65	32.02 ± 15.44	0.14	0.939
Gender (m/f)	22/38	24/36	25/35	23/37	0.35	0.950
Disease duration (years)	3.63 ± 4.34	3.72 ± 4.61	5.26 ± 6.09	4.90 ± 5.55	1.50	0.214
Education (years)	10.43 ± 3.32	11.08 ± 3.35	10.80 ± 2.95	10.32 ± 3.23	0.71	0.545
TRD or not (y/n)	18/42	14/46	15/45	17/43	0.85	0.837
Antidepressants type					3.30	0.993
Escitalopram	30	28	32	30		
Fluoxetine	5	7	7	5		
Sertraline	12	10	8	10		
Venlafaxine	6	8	7	10		
Duloxetine	7	7	6	5		
HDRS-24 score	26.53 ± 5.61	26.28 ± 4.64	25.03 ± 4.02	26.12 ± 5.53	1.05	0.370
RBANS total score	88.82 ± 13.38	90.02 ± 11.68	88.27 ± 13.20	88.25 ± 11.79	0.27	0.844
Immediate memory	84.80 ± 14.79	85.63 ± 16.30	85.58 ± 15.82	82.33 ± 15.38	0.59	0.619
Visuospatial/ constructional	91.28 ± 14.50	93.92 ± 15.03	91.78 ± 15.75	88.50 ± 16.50	1.25	0.294
Language	95.87 ± 11.93	94.07 ± 10.20	91.10 ± 13.42	92.85 ± 14.19	1.54	0.204
Attention	101.28 ± 12.02	100.35 ± 13.06	101.18 ± 12.27	99.23 ± 16.17	0.30	0.827
Delayed memory	88.83 ± 15.28	89.77 ± 13.71	87.20 ± 13.71	86.87 ± 13.20	0.57	0.633

The data are presented as mean ± standard deviation (SD)

*Abbreviation:* *rTMS *Repetitive transcranial magnetic stimulation, *tDCS *Transcranial direct current stimulation, *TRD *Treatment-resistant depression, *HDRS-24 *Hamilton Depression Rating Scale-24, *RBANS *Repeatable Battery for the Assessment of Neuropsychological Status

Before treatment, three-dimensional high-resolution T1 weighted images were acquired by magnetic resonance imaging (MRI) scanning for targeted therapy. The MRI data were collected by a uMR 890 3.0-T magnetic resonance imaging system (Lianying Co Ltd., Shanghai, China). The acquired MRI images were imported into a compatible Brainsight TMS navigation system (Rogue Research Inc., Montreal, Canada). We stimulated targets using the bilateral BA46 definitions, which is considered to be the strongest anti-correlated functional connection between the DLPFC and the subgenual cingulate [17–19]. Based on individual MRI images, the TMS coil or tDCS electrodes were positioned at the designated target location BA46 in the bilateral DLPFC. The stimulated left MNI coordinates were (− 44, 40, 29), whereas the right target had MNI coordinates of (44, 40, 29) [19, 20].

Our tDCS stimulator (Foc.us Ltd., London, UK) provided a constant 2ma DC current via a battery and was used by placing two 5 × 5 cm^2^ sponges moistened with saline on the rubber electrodes. A trained research nurse placed the anode of the tDCS stimulator on the left DLPFC and the cathode on the right DLPFC, followed by 20-min stimulation sessions, 5 times per week (Monday through Friday), for a total of 10 consecutive stimulations over a 2-week period. The device has a sham stimulation mode that rises from 3 s to 2 mA, is maintained for 30 s, and then slowly falls to 0 mA with no stimulation effect. Patients received rTMS within 30–60 min of the end of tDCS stimulation on the same day [21]. Prior to rTMS treatment, the resting motor threshold (RMT) was determined based on the lowest intensity that produced minimal motor evoked potentials (MEPs) greater than 50 mV in the primary motor cortex in 5 consecutive single pulses. A Magstim Rapid2 stimulator (Magstim Co, Ltd, Carmarthenshire, Wales, UK) with a 70-mm-diameter figure-of-eight coil (or equivalent sham coil) was utilized in this study. Stimulation was targeted to the left DLPFC. The add-on TMS group received rTMS treatment with 1600 pulses (4 s on, 26 s off, and 40 repetitions) per day at a frequency of 10 Hz and an RMT intensity of 100% for 2 weeks, 5 times per week. In sham rTMS treatment, the sham magnetic coil looked, sounded, and felt the same as the active rTMS coil used. During this double-blind period, conventional medication remained unchanged and stable in both groups.

All researchers and participants were blinded to treatment assignment during the double-blind phase. Nurses operated only the tDCS or rTMS stimulators. At the end of the study, blinding was assessed by asking participants and raters separately to determine which group the patients were randomly assigned to.

### Clinical assessment and outcomes

The primary outcome measure of cognitive function in these MDD patients was the Repeatable Battery for the Assessment of Neuropsychological Status (RBANS). The total score of the RBANS scale can be used to provide an overall assessment of cognitive function. Our team translated the RBANS into Chinese and established its clinical validity and reliability, which consists of 12 subtests, grouped into five neuropsychological states: attention, language, visuospatial/constructional, immediate memory, and delayed memory, as well as a total score [22, 23]. Two clinical assessments were conducted using the RBANS pre-treatment (baseline) and post-treatment (week 2). A secondary outcome measure was the assessment of clinical symptoms using the HDRS-24, including (1) response rate: defined as a ≥ 50% reduction in HDRS-24 total score from baseline to week 2; (2) change in HDRS-24: reduction in HDRS-24 total score from baseline to week 2; and (3) adverse events.

Trained neuropsychologists conducted all cognitive assessments, and the neuropsychologists were unaware of what interventions the patients had received. The trained neuropsychologists received training on the RBANS and HDRS-24 from a professional scale assessor prior to the formal study. After the training, the neuropsychologists assessed the patients on the RBANS and HDRS-24, and the inter-rater correlation coefficient (ICC) should not be less than 0.8. If it was less than 0.8, the points of disagreement in the scale assessment process were harmonized, and then 2 more patients were approached to undergo the next round of assessment. This rigorous procedure was designed to maintain a high degree of consistency and reliability of the RBANS and HDRS-24 scores, indicating a high degree of agreement between raters throughout the study period.

### Calculation of sample size

We calculated the sample size required to adequately estimate the change in the differences in the RBANS total score between the four groups under a medium effect size of 0.25, power of 80%, and a two-tailed α level of 5%, choosing an *F* test, an ANOVA: repeated measures, and between factor model. The minimum sample size was 136.

### Statistical analysis

SPSS (version 23.0) was used to perform all statistical analyses. Demographic and clinical variables were analyzed using analysis of variance (ANOVA) for continuous variables and chi-square test for categorical variables. Intent-to-treat (ITT) analysis was carried out and missing data were imputed following the mean interpolation.

In this longitudinal study, the effects of four different interventions on patients’ symptoms and cognitive functioning were analyzed in groups A, B, C, and D. The primary outcome was analyzed using repeated measures (RM) multivariate analysis (MANOVA), with two time points (baseline, week 2) as within-group repeated measures and four different intervention groups as between-group repeated measures. If the time and group interaction was significant in the RM MANOVA, post-hot was used to analyze the changes (week 2- baseline) in the four groups using the Bonferroni correction procedure. If the interaction was not significant, no further statistical tests were performed. The same method was used to analyze changes in HDRS-24 scores.

In addition, If the time and group interactions were significant in the HDRS-24 total score, one-way ANOVA was used to compare the changes and also using the Bonferroni correction in four groups. chi-square test was used to compare the differences in the proportions of clinical responders between the groups. Besides, when unblinding at the end of the clinical trial, we used a chi-square test to compare the proportion of patients in each group who correctly guessed the stimulus they received, and to compare the proportion of the type of stimulus patients correctly received to the type of stimulus guessed by raters in each group. All statistical tests were two-tailed and considered statistically significant if the p-value was less than or equal to 0.05.

## Results

### Demographic and basic descriptive data

As shown in Table 1, there were no statistically significant differences between the four groups of patients in terms of demographic characteristics and general clinical variables, including HDRS-24 and RBANS total and domain scores.

A total of 550 patients were recruited during the intervention period, of whom 203 completed the 2-week maintenance intervention (Fig. 1). In addition, reasons for dropout during the treatment period included: In group A _active rTMS + active tDCS_, 5 patients were unable to complete the RBANS scale well, 2 patients returned to school due to exams and 5 patients had no specific reasons. In group B _active rTMS + sham tDCS_, 5 patients could not complete the RBANS scale well, 2 patients did not want to continue treatment and 1 patient considered treatment ineffective. In group C _sham rTMS + active tDCS_, 4 patients were unable to complete the RBANS scale well, and 6 patients dropped out because of itching, but the itching symptom had been relieved by themselves without specific treatment. In group D _sham rTMS + sham tDCS_, 3 patients dropped out because they were unable to complete the RBANS scale well and 4 patients withdrew for no particular reason.

### Integrity of blinding

In fact, at the end of the treatment, we conducted two unblinding tests, both of which were conducted by professionals who retained the blinding background. For the first unblinding, only the group to which each case belonged was listed (e.g., group 1, 2, 3, or 4). Subsequently, a second unblinding was performed to determine which active treatment group and control group corresponded to groups 1, 2, 3, or 4, respectively.

In addition, we asked participants and raters separately whether they were aware of the true stimulus situation. For patients, they were asked if they knew which type of stimuli they received during the treatment. 77.08% (37/48) of patients in group A, 69.23% (36/52) of patients in group B, 70.00% (35/50) of patients in group C, and 58.49% (31/53) of patients in Group D believed that they had received the actual stimulus and felt improved. The chi-square test showed there was no significant difference between the four groups in the proportion of patients who correctly guessed which stimulus they received correctly (*χ* ^2^ = 4.16, *p* = 0.224). At the same time, raters were asked to guess which type of stimulation the patients received. Raters correctly identified 27 patients who received two active stimuli (27/48), 29 patients who received active rTMS in the active rTMS + sham tDCS (29/52), 17 patients who received active tDCS in the sham rTMS + active tDCS (17/50) and 23 patients who received two sham stimulations (23/53) in the sham rTMS + sham tDCS group. The results of the chi-square test showed that there was no significant difference in the proportion of raters who correctly guessed the type of stimulus each patient received (*χ* ^2^ = 6.91, *p* = 0.075).

### Efficacy of combination therapy on cognitive performance

The RBANS total and index scores showed a significant group × time interaction (*F*_(3,236)_ = 11.59, *p* < 0.001, *η*2 = 0.13) as well as a time effect (*F*_(1,236)_ = 74.50, *p* < 0.001, *η*2 = 0.240) rather than a group effect (*F*_(3,236)_ = 2.60, *p* = 0.053, *η*2 = 0.032). In addition, the RM ANCOVA for the 5 RBANS domains revealed a significant time effect (*F*_(1,236)_ = 117.27, *p* < 0.001, *η*2 = 0.330) on Immediate memory score, as well as a significant group-by-time interaction (*F*_(3,236)_ = 3.46, *p* = 0.017, *η*2 = 0.042), but no significant group effect (*F*_(3,236)_ = 2.09, *p* = 0.102, *η*2 = 0.026). The RM ANCOVA for Visuospatial/Constructional score also revealed a significant group-by-time interaction effect (*F*_(3, 236)_ = 6.75, *p* < 0.001, *η*2 = 0.079), together with a significant time effect (*F*_(1, 236)_ = 23.83, *p* < 0.001, *η*2 = 0.092). Table 2 presents the results of the RM ANCOVA for other individual domains of RBANS.

**Table 2:** Cognitive score and comparison at baseline and week 2 in four groups

	Baseline	Week 2	Group F (*P* value)	Time F (*P* value)	Group * time (*P* value)
**RBANS total score**			2.60 (0.053)	74.50 (0.000)	11.59 (0.000)
Real rTMS + real tDCS	88.81 ± 12.38	98.56 ± 12.63			
Real rTMS + sham tDCS	90.01 ± 11.68	95.42 ± 11.38			
Sham rTMS + real tDCS	88.27 ± 13.20	92.66 ± 12.69			
Sham rTMS + sham tDCS	88.25 ± 11.79	88.49 ± 12.24			
**Immediate memory**			2.09 (0.102)	117.27 (0.000)	3.46 (0.017)
Real rTMS + real tDCS	84.80 ± 14.79	98.21 ± 16.79			
Real rTMS + sham tDCS	85.63 ± 16.30	94.48 ± 16.68			
Sham rTMS + real tDCS	85.58 ± 15.82	94.32 ± 17.28			
Sham rTMS + sham tDCS	82.33 ± 15.38	88.09 ± 15.37			
**Visuospatial/constructional**			2.21 (0.087)	23.83 (0.000)	6.75 (0.000)
Real rTMS + real tDCS	91.28 ± 14.50	99.42 ± 15.21			
Real rTMS + sham tDCS	93.92 ± 15.03	94.98 ± 14.73			
Sham rTMS + real tDCS	91.78 ± 15.75	94.54 ± 16.35			
Sham rTMS + sham tDCS	88.50 ± 16.50	89.39 ± 16.25			
**Attention**			1.16 (0.326)	4.15 (0.043)	1.93 (0.000)
Real rTMS + real tDCS	95.87 ± 11.93	96.48 ± 10.67			
Real rTMS + sham tDCS	94.07 ± 10.20	94.75 ± 10.12			
Sham rTMS + real tDCS	91.10 ± 13.42	95.34 ± 12.65			
Sham rTMS + sham tDCS	92.85 ± 14.19	92.92 ± 11.66			
**Language**			0.44 (0.724)	5.12 (0.025)	0.059 (0.981)
Real rTMS + real tDCS	101.28 ± 12.02	103.46 ± 10.42			
Real rTMS + sham tDCS	100.35 ± 13.06	101.63 ± 11.72			
Sham rTMS + real tDCS	101.18 ± 12.27	102.84 ± 12.90			
Sham rTMS + sham tDCS	99.23 ± 16.17	101.09 ± 15.50			
**Delayed memory**			0.442 (0.723)	59.15 (0.000)	1.47 (0.223)
Real rTMS + real tDCS	88.83 ± 15.28	93.17 ± 13.85			
Real rTMS + sham tDCS	89.77 ± 13.75	93.31 ± 12.51			
Sham rTMS + real tDCS	87.20 ± 13.71	94.26 ± 11.98			
Sham rTMS + sham tDCS	86.87 ± 13.20	91.32 ± 10.83			

The data are presented as mean ± standard deviation (SD)

*rTMS *Repetitive transcranial magnetic stimulation, *tDCS *Transcranial direct current stimulation, *RBANS *Repeatable Battery for the Assessment of Neuropsychological Status

After 2 weeks of treatment, there was a significant difference in the change in RBANS total scores of the four groups compared to baseline (*F* = 11.60, *p* < 0.001, *η*2 = 0.128). Interestingly, post-hoc tests showed a significant increase in the RBANS total score in the combined treatment group compared to the other three groups (*p* = 0.048 (group A vs. group B); *p* = 0.007 (group A vs. group C); *p* < 0.001 (group A vs. group D). The results also showed a significant increase in the RBANS total score in the active rTMS group compared to the sham group (*p* = 0.01 (group B vs. group D)), whereas there was no significant difference between the active rTMS and active tDCS group (*p* = 0.833). However, there was also no significant difference between the active tDCS group and the sham group (*p* = 0.066).

The same results were noted for immediate memory (*F* = 3.46, *p* = 0.017, *η*2 = 0.042) and visuospatial/constructional indices (*F* = 6.75, *p* < 0.001, *η*2 = 0.079), with statistically significant differences between the four groups. Interestingly, the combined treatment group showed better improvement than the sham treatment group in terms of the immediate memory index (*p* = 0.01 (group A vs. group D)), but in terms of the visuospatial/constructional index, the combined treatment group had a significant advantage over the other three groups (*p* < 0.001 (group A vs. group B); *p* = 0.024 (group A vs. group C); and *p* < 0.001 (group A vs. group D)).

However, with respect to the immediate memory index and the visuospatial/constructional index, there was no significant difference between the sham group and either the active rTMS group (all *p* > 0.05 (group D vs. group B)) or the active tDCS group (all *p* > 0.05 (group D vs. group C)). Also, there was no significant difference between the active rTMS group and the active tDCS group (all *p* > 0.05 (group B vs. group C)).

### Combined treatment of depressive symptoms

The HDRS-24 total scores for the four groups showed a significant group-by-time interaction effect (*F*_(3,236)_ = 10.49, *p* < 0.001, *η*2 = 0.118) as well as a time effect (*F*_(1,236)_ = 1687.43 *p* < 0.001, *η*2 = 0.877) but group effect failed (*F*_(3,236)_ = 2.52, *p* = 0.059, *η*2 = 0.031) (Table 3).

**Table 3:** HDRS-24 scores at baseline and week 2 in four groups

	Baseline	Week 2	Group F (*P* value)	Time F (*P* value)	Group * Time (*P* value)
HDRS-24 score			2.52 (0.059)	1687.43 (0.000)	10.49 (0.000)
Active rTMS + active tDCS (*N* = 60)	26.53 ± 5.61	9.94 ± 3.65			
Active rTMS + sham tDCS (*N* = 60)	26.28 ± 4.64	11.12 ± 5.84			
Sham rTMS + active tDCS (*N* = 60)	25.03 ± 4.02	12.28 ± 4.81^b*^			
Sham rTMS + sham tDCS (*N* = 60)	26.12 ± 5.53	14.38 ± 5.89^a*^			

The data are presented as mean ± standard deviation (SD)

*rTMS*, repetitive transcranial magnetic stimulation; *tDCS*, transcranial direct current stimulation; *HDRS-24*, Hamilton Depression Rating Scale-24

^a^Indicates the comparison between active rTMS + active tDCS and sham rTMS + sham tDCS in HDRS-24 scores change, *p < 0.05

^b^Indicates the comparison between active rTMS + active tDCS and sham rTMS + active tDCS in HDRS-24 scores change, *p < 0.05

The HDRS-24 total score was significantly lower in all four groups after 2 weeks of treatment (group A _active rTMS + active tDCS_ = 16.00, group B _active rTMS + sham tDCS_ = 15.17, group C _sham rTMS + active tDCS_ = 12.75, and group D _sham rTMS + sham tDCS_ = 11.73, *F* = 10.49, *p* < 0.001, *η*2 = 0.118). Interestingly, post-hoc tests showed that the HDRS-24 total score was significantly lower in the combined treatment group than in the active tDCS group (*p* = 0.001 (group A vs. group C)) and sham group (*p* < 0.001 (group A vs. group D)). We also found a significant difference in the change in HDRS-24 score between the active rTMS group and tDCS (*p* = 0.013 (group B vs. group C)) or active rTMS group and sham group (*p* < 0.001 (group B vs. group D)). The chi-square tests revealed a significant difference in response rates among the four groups (*χ* ^2^ = 14.18, *p* = 0.003). 83.33% response rate was observed in group A _active rTMS + active tDCS,_ which was higher than that in group B _active rTMS + sham tDCS_ (71.15%), group C _sham rTMS + active tDCS_ (62.00%), or group D _sham rTMS + sham tDCS_ (49.05%) (all *p* < 0.05).

### Adverse events and safety

All patients tolerated the treatment well, with no serious adverse reactions and no statistical difference in the incidence of adverse events between the four groups. Common adverse reactions included skin redness (1 case in group A _active rTMS + active tDCS_, 2 cases in group B _active rTMS + sham tDCS_, 3 cases in group C _sham rTMS + active tDCS_ and 3 cases in group D _sham rTMS + sham tDCS_), dizziness (1 case in group A _active rTMS + active tDCS_ and 2 cases in group C _sham rTMS + active tDCS_), pruritus (2 cases in group A _active rTMS + active tDCS_, 3 cases in group C _sham rTMS + active tDCS_ and 2 cases in group D _sham rTMS + sham tDCS_), nausea (1 case in group C _sham rTMS + active tDCS_), mild irritation(3 cases in group C _sham rTMS + active tDCS_), insomnia (2 cases in group B _active rTMS + sham tDCS_ and 2 cases in group D _sham rTMS + sham tDCS_), and headache (3 cases in group A _active rTMS + active tDCS_ and 2 cases in group B _active rTMS + sham tDCS_). Most of these mild adverse reactions resolved within 2 h of the end of the intervention, and slightly more severe effects resolved on their own within 2 days without the need for additional treatment. There were no significant changes in vital signs throughout the study. No patients experienced seizures or manic symptoms.

## Discussion

This randomized, controlled, double-blind study demonstrated for the first time that the combination of rTMS and tDCS therapy improved cognitive deficits in MDD patients, particularly in the RBANS total score, immediate memory, and visuospatial/constructional index. Moreover, rTMS and tDCS therapy significantly increased the RBANS total score and visuospatial/constructional compared to the other three groups but only showed a better improvement than the sham group in Immediate memory. In addition, the results also showed increased RBANS total score significantly higher in the active rTMS group compared with the sham group. Unexpectedly, the results of this study showed that patients treated with tDCS or rTMS alone did not show better than other cognitive performance compared to the sham group. In addition, We also found that the combination treatment reduced depressive symptoms more rapidly than tDCS or sham alone and rTMS alone is better than the sham group.

Both rTMS and tDCS are widely used antidepressant approaches [24, 25], and our results showed that both rTMS and tDCS improved clinical symptoms, which is consistent with previous studies [26–28]. The antidepressant mechanisms of rTMS and tDCS are highly correlated with neurochemical systems, as well as baseline abnormal brain activity [29, 30]. For example, MDD is characterized by asymmetry between the two prefrontal regions, with elevated metabolic and neuronal activity on the right side and decreased activity on the left side [31, 32]. Studies have demonstrated that anodal tDCS stimulation and high-frequency rTMS increase cortical excitability, whereas cathodal tDCS stimulation and low-frequency rTMS decrease cortical activity [21, 33, 34]. Therefore, high-frequency rTMS and anodal tDCS over L-DLPFC are efficacious for MDD. Additionally, participants who received the combination of rTMS and tDCS showed the greatest antidepressant effect, which may be mainly due to the pre-treatment with tDCS that alters the resting membrane potential of neurons, resulting in a stronger therapeutic effect of rTMS.

Previous studies have shown inconsistent results of rTMS on cognitive improvement [35]. Our current study showed that although rTMS alone reduced the HDRS-24 total score of patients with MDD to a greater extent, there was also a statistically significant difference between the rTMS-only group and the sham group in terms of cognitive improvement. Potential pro-cognitive mechanisms of rTMS may be associated with direct changes in intraregional activity or the level of connected neural networks [36]. For example, rTMS improves cognitive function due to the release of dopamine from the caudate nucleus and striatum, as well as an increase in functional neuroanatomical connections between the DLPFC and subcortical structures such as the anterior cingulate cortex (ACC) and striatum [37]. In addition, rTMS has been found to increase the neural efficiency of cognitive processing speed and cortical plasticity, which may contribute to accelerating cognitive processes [38, 39].

Although tDCS alone did not show better cognitive improvements than the sham group. The fact suggested that tDCS has the potential to improve cognition. However, it needs to be more focused on targeting localized areas. Therefore, compared with rTMS, the facilitation effect of tDCS was more limited, and the trial results showed greater variability [40]. The mechanism of cognitive facilitation by prefrontal tDCS is similar to that of rTMS, with effects on both the targeted areas and the connected neural networks. For example, studies have reported that tDCS applied to the DLPFC increases striatal dopamine release [41] or modulates dopamine-GABA function in the basal ganglia-cortical circuit [42]. Dopamine is associated with neuronal efficiency and higher-order cognitive processes such as working memory and learning in the striatum and prefrontal cortex [43, 44]. Meta-analyses have indicated that tDCS also increases cortical activation, with fNIRS signaling increasing after stimulation in both healthy adults and psychiatric patients [45, 46].

In our study, a 10-day combination of tDCS and rTMS treatment for left DLPFC showed a significant improvement in the RBANS total score than active rTMS or tDCS treatment. On the one hand, ten days of tDCS or rTMS treatment alone may not be sufficient, and although rTMS combined tDCS therapy has not been tested in MDD patients, it has been tested in other neuropsychiatric disorders. For example, in patients with Parkinson’s disease, rTMS combined with tDCS treatment was significantly superior to rTMS alone in improving executive function [45]. Similarly, in patients recovering from stroke, the combination of rTMS and tDCS treatment was superior to rTMS alone in improving motor function [16, 47, 48]. Therefore, we hypothesize that rTMS combined tDCS treatment is more effective than rTMS or tDCS alone, possibly through molecular effects that alter intrinsic and extrinsic properties of neurons, mediate the expression of neurotransmitters and their receptors as well as the activation of neurotrophic factors, and lead to long-term changes in synaptic plasticity, producing faster and better behavioral performance [49].

Multiple factors contributed to the improvement in neurocognitive function with the combination therapy, including the fact that tDCS has a preconditioning effect. When patients receive anodal tDCS on the left DLPFC, neuronal depolarization and cortical excitability increase, while inducing long-term potentiation of neurons and altering brain plasticity [26]. With tDCS treatment followed by rTMS, anode-induced neuronal depolarization leads to the direct generation of action potentials in cortical neurons. The combination of the two treatments maximizes the therapeutic effect of rTMS. Patients who receive rTMS in the left DLPFC after tDCS treatment may experience more substantial brain changes. Other studies on combination therapies have also shown better efficacy. For example, a study combining D-cycloserine with iTBS found that the use of a low dose of the NMDA receptor agonist D-cycloserine resulted in longer-lasting iTBS-induced plasticity effects and greater symptomatic improvement in depressed patients [50]. Another combination therapy of bright light therapy with fluoxetine in patients with non-seasonal MDD showed that both monotherapy and combination therapy with fluoxetine were effective and well tolerated in the treatment of non-seasonal MDD. However, the combination therapy was the most effective [51]. In addition, the frequency, overall duration, and dose of daily treatments appear to be related to the efficacy of either therapy alone. Using one of the therapies multiple times per day may enhance the therapeutic effect [52, 53].

We found a significant increase in the RBANS visuospatial score of rTMS combined with tDCS treatment compared to the other three groups. Prefrontal rTMS combined with tDCS treatment may exert cognitive effects through neuroplasticity in the visual cortex. A study by Zhang and his colleague found in both clinical and animal experiments that using magnetic stimulation to intervene in the primary visual cortex (V1) can significantly improve depressive-like behavior, the alleviation of depression was associated with altered synaptic plasticity in the ABCA1/ApoA1 signaling pathway in V1 [54]. In addition, rTMS given to the visual cortex (VC) effectively alleviates clinical symptoms in MDD patients. Moreover, symptom reduction was correlated with improvements in functional MRI during the performance of VC tasks [55]. Meta-analysis has indicated that rTMS produces specific modest enhancements in visual scanning ability, suggesting that the VC may be a potential target for the treatment of MDD symptoms [6].

rTMS combined with tDCS treatment also showed significantly better improvement in immediate memory score than the other three groups, but there was no significant difference between the active rTMS, tDCS, and sham groups. This is also consistent with previous studies, where a large sample multicenter randomized sham-controlled trial on cognitive effects also found that although stimulation therapy with a 3-week intervention on left DLPFC, there was no statistically significant difference between 10 Hz rTMS and sham groups [56]. Another study on tDCS showed that stimulation of the DLPFC with tDCS for 2 weeks (10 sessions) had no significant effect on working memory and overall cognition in depressed patients compared to a sham intervention [57]. rTMS combined with tDCS treatment significantly improved immediate memory, which may be due to the fact that tDCS combined with rTMS stimulation enhances neurogenesis, thereby increasing the concentration of neurotransmitters, including N-Methyl-D-Aspartate (NMDA) and brain-derived neurotrophic factor (BDNF), leading to synaptic plasticity-related changes, such as long-term potentiation [58, 59]. Another reason is that tDCS combined with rTMS stimulates the DLPFC, activating brain regions such as the anterior cingulate gyrus, amygdala, hippocampus, and orbitofrontal lobe, and enhancing connections between brain regions that have been shown to be important for various cognitive processes [60, 61]. However, further research is needed to determine the exact mechanism by which rTMS combined with tDCS is effective in improving cognitive impairment.

There are some limitations to this study. First, this trial only assessed outcomes over a short period of time and there was no follow-up. Second, we did not control for the effects of antidepressant medications, and therefore some patients may have experienced some confounding effects of antidepressants. Third, for a number of practical reasons, we did not strictly follow the original protocol. Although the ethics committee agreed to changes and approved our new protocol, we should plan more accurately and follow the protocol strictly when conducting similar clinical studies in the future. In addition, the lack of exploration of biological and neural mechanisms in this study prevented us from accurately determining the mechanisms of the combination treatment. In the future, joint exploration with electrophysiology, MRI, and blood biomarkers will be considered. Future studies need to investigate longer courses of the combination of tDCS and rTMS in larger samples, and more uniform antidepressant use.

## Conclusions

In summary, this 10-day double-blind, sham-controlled randomized clinical trial demonstrated that rTMS combined with tDCS treatment significantly improved clinical symptoms and increased immediate memory, visuospatial/constructional domain, and RBANS total scores in MDD patients over a shorter treatment period compared with sham treatment. However, the results of this study should be confirmed in future studies of longer courses of tDCS combined with rTMS treatment in larger samples of MDD patients from other ethnic groups.

## Data Availability

The datasets used and analyzed during the current study are available from the corresponding author on reasonable request.

## Abbreviations

ACC: Anterior cingulate cortex
BDNF: Brain-derived neurotrophic factor
DLPFC: Dorsolateral prefrontal cortex
DSM-V: Diagnostic and Statistical Manual of Mental Disorders-V
HDRS-24: 24-Item Hamilton Depression Rating Scale
ICC: Inter-rater correlation coefficient
ITT: Intent-to-treat
MANOVA: Multivariate analysis
MDD: Major depressive disorder
MEPs: Motor evoked potentials
MRI: Magnetic resonance imaging
NMDA: N-Methyl-D-Aspartate
RBANS: Repeatable Battery for the Assessment of Neuropsychological Status
RMT: Resting motor threshold
rTMS: Repetitive transcranial magnetic stimulation
tDCS: Transcranial direct current stimulation
VC: Visual cortex
